# Molecular Mechanism of Macrophage Activation by Red Ginseng Acidic Polysaccharide from Korean Red Ginseng

**DOI:** 10.1155/2012/732860

**Published:** 2012-02-01

**Authors:** Se Eun Byeon, Jaehwi Lee, Ji Hye Kim, Woo Seok Yang, Yi-Seong Kwak, Sun Young Kim, Eui Su Choung, Man Hee Rhee, Jae Youl Cho

**Affiliations:** ^1^Department of Genetic Engineering, Sungkyunkwan University, Suwon 440-746, Republic of Korea; ^2^College of Pharmacy, Chung-Ang University, Seoul 122-704, Republic of Korea; ^3^Korea Ginseng Corporation, Central Research Institute, Daejeon 305-805, Republic of Korea; ^4^Department of Efficacy Screening, Hongcheon Institute of Medicinal Herb, Hongcheon 250-930, Republic of Korea; ^5^Department of Natural Product Resources, DanjoungBio, Wonju 220-842, Republic of Korea; ^6^College of Veterinary Medicine, Kyungpook National University, Daegu 702-701, Republic of Korea

## Abstract

Red ginseng acidic polysaccharide (RGAP), isolated from Korean red ginseng, displays immunostimulatory and antitumor activities. Even though numerous studies have been reported, the mechanism as to how RGAP is able to stimulate the immune response is not clear. In this study, we aimed to explore the mechanism of molecular activation of RGAP in macrophages. RGAP treatment strongly induced NO production in RAW264.7 cells without altering morphological changes, although the activity was not strong compared to LPS-induced dendritic-like morphology in RAW264.7 cells. RGAP-induced NO production was accompanied with enhanced mRNA levels of iNOS and increases in nuclear transcription factors such as NF-**κ**B, AP-1, STAT-1, ATF-2, and CREB. According to pharmacological evaluation with specific enzyme inhibitors, Western blot analysis of intracellular signaling proteins and inhibitory pattern using blocking antibodies, ERK, and JNK were found to be the most important signaling enzymes compared to LPS signaling cascade. Further, TLR2 seems to be a target surface receptor of RGAP. Lastly, macrophages isolated from RGS2 knockout mice or wortmannin exposure strongly upregulated RGAP-treated NO production. Therefore, our results suggest that RGAP can activate macrophage function through activation of transcription factors such as NF-**κ**B and AP-1 and their upstream signaling enzymes such as ERK and JNK.

## 1. Introduction

Korean ginseng (the root of *Panax ginseng* C.A. Meyer) is a representative herbal which is ethnopharmacologically well known in East Asian countries, including Korea, China, and Japan for about 2,000 years. This plant is indeed widely used as a supplementary herbal medicine for treating numerous diseases such as cancer, diabetes, and atherosclerosis [1]. Active constituents of ginseng are reported to be ginsenosides, acid polysaccharides, peptides, polyacetylenic alcohols, and fatty acids [2]. Of these ingredients, ginsenosides have been known as the major active compounds with a variety of pharmacological activities such as antidiabetic, anticancer, and antiinflammatory effects [2–6]. In contrast to the ginsenosides, pharmacological efficacy of the polysaccharide fractions has not been fully investigated. 

Nonetheless, several studies have demonstrated that immunostimulatory functions of red ginseng could be due to red ginseng acid polysaccharide (RGAP) [2]. Thus, it has been stressed that acid polysaccharides from the root of *Panax ginseng* play a critical role in displaying mitogenic, antitumor, and direct immunostimulating activities in cyclophosphamide-treated immunosuppressed mice [2, 7–9]. RGAP was reported to upregulate the functional roles of natural killer cells and macrophages linked to antitumor activities [10, 11]. Furthermore, this polysaccharide has been found to diminish the incidence rate of benzo[a]pyrene-mediated neoplasms [12].

Although previous papers indicated its immunostimulatory roles in various immune cells, the exact molecular mechanism of RGAP in macrophages has not been fully elucidated. In this study, therefore, we aimed to explore how RGAP can stimulate functional activation of macrophages by measuring molecular events and characterizing surface receptors and also understand how the immunostimulatory activity by RGAP occurs. 

## 2. Materials and Methods

### 2.1. Materials

RGAP isolated from Korean red ginseng was performed by steaming and drying fresh ginseng root (*Panax ginseng* C.A. Meyer) as described previously [13, 14] and was kindly supplied by the Korea Ginseng Corporation (Daejeon, Republic of Korea). (3-4,5-Dimethylthiazol-2-yl)-2,5-diphenyltetrazolium bromide, a tetrazole (MTT), and lipopolysaccharide (LPS, *E. coli* 0111:B4) were purchased from Sigma Chemical Co. (St. Louis, MO). Piceatannol, SP600125, U0126, PP2, and pam3CSK were obtained from Calbiochem (La Jolla, CA). *β*-glucan was purified from *Lentinus edodes* [15]. Foetal bovine serum and RPMI 1640 were obtained from GIBCO (Grand Island, NY). RAW264.7 cells were purchased from ATCC (Rockville, MD). All other chemicals were of Sigma grade. Phosphospecific or total antibodies to p65, c-fos, c-Jun, CREB, extracellular signal-related kinase (ERK), c-Jun N-terminal kinase (JNK), p38, Akt, I*κ*B*α*,*γ*-tubulin, *β*-tubulin, and *β*-actin were obtained from Cell Signaling (Beverly, MA). 

### 2.2. Animals

RGS2 knockout mice [16] were kindly supplied from Dr. Blumber (Washington University, St. Louis, MO). Wild-type C57BL/6 male mice (6–8 weeks old, 17–21 g) were obtained from DAEHAN BIOLINK (Chungbuk, Republic of Korea) and maintained in plastic cages under conventional conditions. Water and pellet diets (Samyang, Daejeon, Republic of Korea) were available *ad libitum*. Studies were performed in accordance with guidelines established by the Kangwon University Institutional Animal Care and Use Committee.

### 2.3. Preparation of Peritoneal Macrophage

Peritoneal exudates were obtained from wild-type or RGS-2 knockout C57BL/6 male mice (7-8 weeks old, 17–21 g) by lavaging 4 days after intraperitoneal injection of 1 mL of sterile 4% thioglycollate broth (Difco Laboratories, Detroit, MI) as reported previously [17, 18]. After washing with RPMI 1640 medium containing 2% FBS, peritoneal macrophages (1 × 10^6^ cells/mL) were plated in 100 mm tissue culture dishes for 4 h at 37°C in a 5% CO_2_ humidified atmosphere.

### 2.4. Cell Culture

Peritoneal macrophages and RAW264.7 cells were cultured with RPMI 1640 medium supplemented with 10% heat-inactivated fetal bovine serum (Gibco, Grand Island, NY), glutamine, and antibiotics (penicillin and streptomycin) at 37°C under 5% CO_2_. For each experiment, cells were detached with a cell scraper. Under our experimental cell density (2 × 10^6^ cells/mL), the proportion of dead cells was less than 1%, according to Trypan blue dye exclusion tests. 

### 2.5. NO Production

After preincubation of RAW264.7 cells (1×10^6^ cells/mL) for 18 h, cells were treated with RGAP (0 to 4 mg/mL) or LPS (1 *μ*g/mL) for 24 h. The inductive effect of RGAP on NO production was determined by analyzing NO levels with the Griess reagent, as described previously [19, 20].

### 2.6. mRNA Analysis by Semiquantitative Reverse Transcriptase-Polymerase Chain Reaction (RT-PCR)

To evaluate iNOS mRNA expression levels, total RNA was isolated from LPS-treated RAW264.7 cells with TRIzol Reagent (Gibco BRL), according to the manufacturer's instructions. The total RNA was stored at −70°C until use. Semiquantitative RT reactions were conducted as reported previously [21, 22]. The primers (Bioneer, Seoul, Republic of Korea) used are indicated in Table 1.

### 2.7. Preparation of Total Lysates and Nuclear Extracts and Immunoblotting

Preparation of total lysates and nuclear extracts from LPS-treated RAW264.7 cells pretreated with RGAP or LPS was done using a method previously published [23, 24]. Immunoblotting of phosphorylated or total levels of transcription factors (AP-1 and p65), MAPK (ERK, p38, and JNK), I*κ*B*α*, IKK*β*, Akt, p85/PI3K, PDK1, *γ*-tubulin, and nonreceptor tyrosine kinases (Src and Syk) was done according to previously published methods [25].

### 2.8. Statistical Analysis

**Figure 1:**
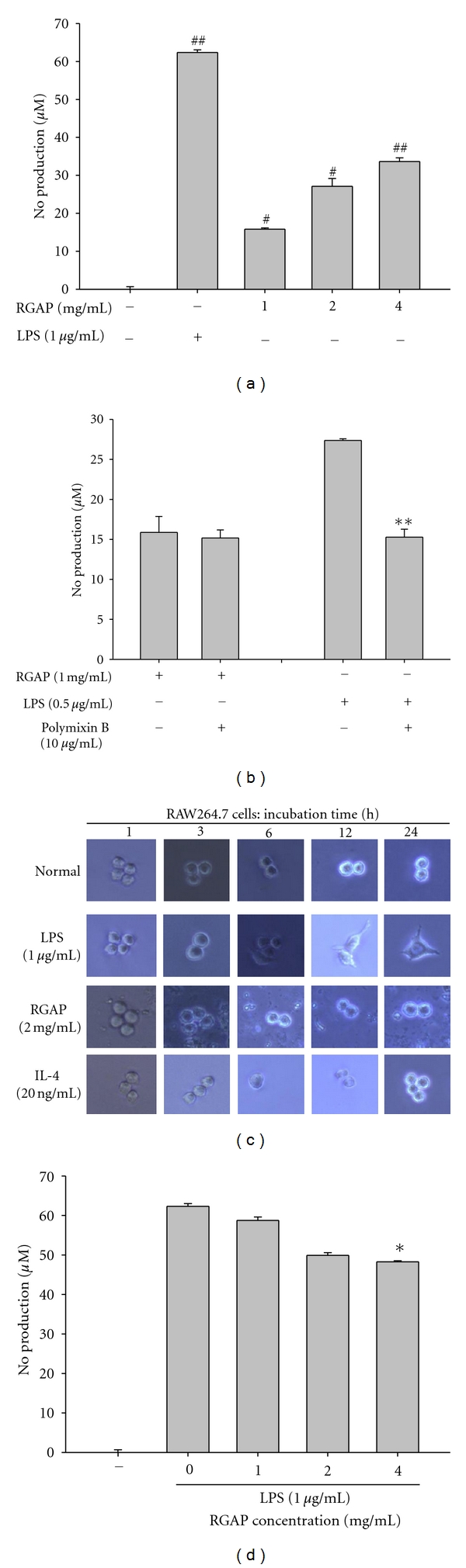
Effects of RGAP and LPS on production of NO and morphological changes. (a, b, and d) Levels of NO were determined by Griess assay from culture supernatants of RAW264.7 cells treated with RGAP and LPS (1 *μ*g/mL) for 24 h. (c) Morphological changes in RAW264.7 cells treated with LPS (1 *μ*g/mL), RGAP (2 mg/mL), and IL-4 (20 ng/mL). Images were taken by a digital camera. ^#^ *P* < 0.05 and ^##^ *P* < 0.01 compared to normal and *P < 0.05 and **P < 0.01 compared to control.

**Figure 2:**
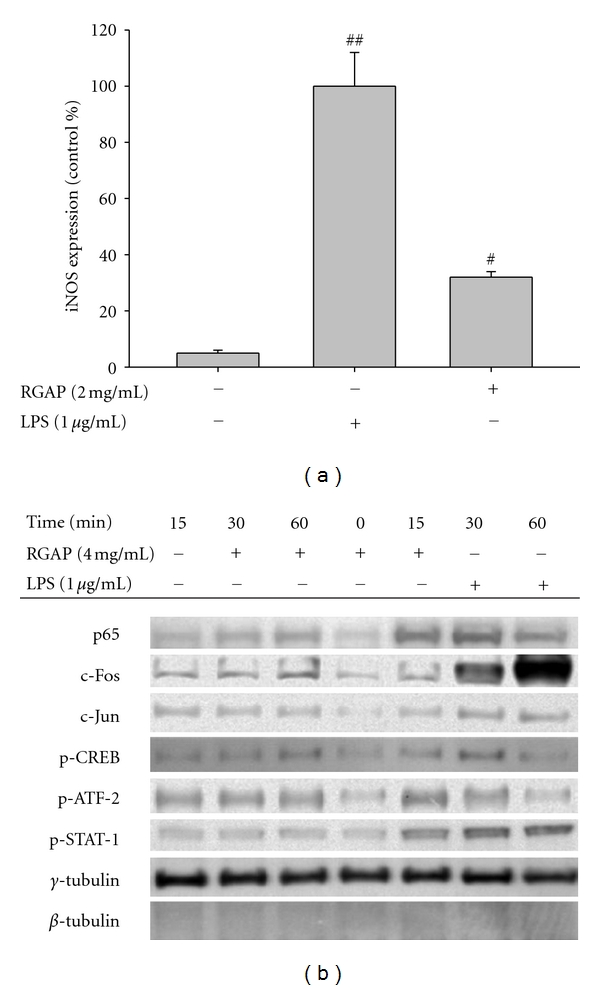
Effect of RGAP on iNOS mRNA expression of and activation of transcription factors. (a) The mRNA levels of iNOS and GAPDH were determined by real-time PCR. (b) Total or phosphorylated levels of transcription factors (NF-*κ*B (p65), AP-1 (c-Fos and c-Jun)), CREB, ATF-2, STAT-1, *γ*-tubulin, and *β*-tubulin in nuclear fractions were determined by immunoblotting analysis with antibodies against the total or phosphorylated proteins. ^#^ *P* < 0.05 and ^##^ *P* < 0.01 compared to normal.

**Figure 3:**
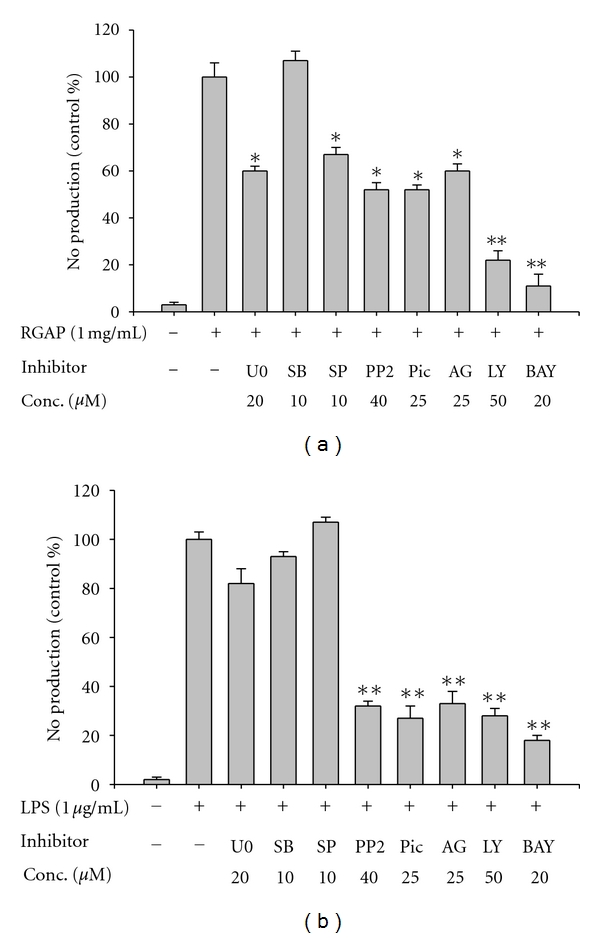
Effects of enzyme inhibitors on RGAP- or LPS-mediated NO production in RAW264.7 cells. (a and b) Levels of NO were determined by the Griess assay from culture supernatants of RAW264.7 cells pretreated with MAPK inhibitors (U0126 (U0), SB203580 (SB), and SP600125 (SP)), tyrosine kinase inhibitors (PP2, piceatannol (Pic), and AG126 (AG)), LY294002 (LY), and BAY11-7082 (BAY), after RGAP (1 mg/mL) or LPS (1 *μ*g/mL) treatment for 24 h. *P < 0.05 and **P < 0.01 compared to control.

Data (Figures 1(a), 1(b), 1(d), 2(a), 3, 5, and 6), expressed as means ± standard deviations (SD), were calculated from at least three independent experiments, each performed in triplicate. Other data are representative of three different experiments with similar results. For statistical comparisons, results were analyzed using analysis of variance/Scheffe's post hoc test and a Kruskal-Wallis/Mann-Whitney test. A *P* < 0.05 was considered a statistically significant difference. All statistical tests were carried out using the computer program SPSS (SPSS Inc., Chicago, IL).

## 3. Results and Discussion

Polysaccharides isolated from basidiomycetes have been reported to act as immunostimulators. The fungal polysaccharides (e.g., lentinan) originating from *Lentinus edodes* is composed of the basic structure of a *β*-1,3-glucan with *β*-1,6-glucopyranosidic branches and has also showed immunostimulating properties [26–28]. In contrast, RGAP has also been found to have immunostimulating and antitumor activities in tumor-bearing models [11], but the exact mechanism of these effects was not fully investigated. As continuous work, therefore, the immunostimulatory mechanism of RGAP was evaluated using macrophages.

Our previous data indicate that RGAP was capable of modulating functional activation of macrophages [28]. Thus, this polysaccharide (0 to 4 mg/mL) significantly induced NO production, although such induction level was not higher than LPS exposure (Figure 1(a)) or comparable with other immunogens such as poly-*γ*-glutamic acid, lactic acid bacterium-derived peptidoglycans, and *β*-glucans [29, 30]. RGAP-induced NO production seemed not to be due to the contamination of endotoxin, since NO release by RGAP was not blocked with polymixin B (Figure 1(b)), a cyclic polycationic peptide antibiotic that binds to anionic lipids such as LPS [31]. Interestingly, the activation pattern of macrophages by LPS (1 *μ*g/mL) was distinct from that of RGAP (2 mg/mL). For example, the morphological change of macrophages observed by LPS exposure was not induced by RGAP and IL-4 (Figure 1(c)). Furthermore, higher concentrations of RGAP (4 mg/mL) antagonized LPS-induced production up to 18% (Figure 1(d)), suggesting that the activation mechanism between LPS and RGAP could be different. 

To confirm whether the production of NO by RGAP is managed by cellular transcription factor activation, levels of iNOS expression and transcription factors required for iNOS gene expression were explored. As Figure 2(a) indicates, iNOS expression showed similar pattern in both groups, but RGAP-induced iNOS gene expression was much lower than LPS group. In particular, the nuclear translocation levels of phosphorylated or total transcription factors such as NF-*κ*B (p65), AP-1 (c-Jun and c-Fos), CREB, ATF-2, and STAT-1 required for iNOS promoter activity [32] was also seen in both groups. Similar results showing that RGAP was able to stimulate NF-*κ*B activation in macrophages have been reported previously [33]. However, the levels of these factors were clearly lowered in the RGAP-treated groups (Figure 2(b)).

**Figure 4:**
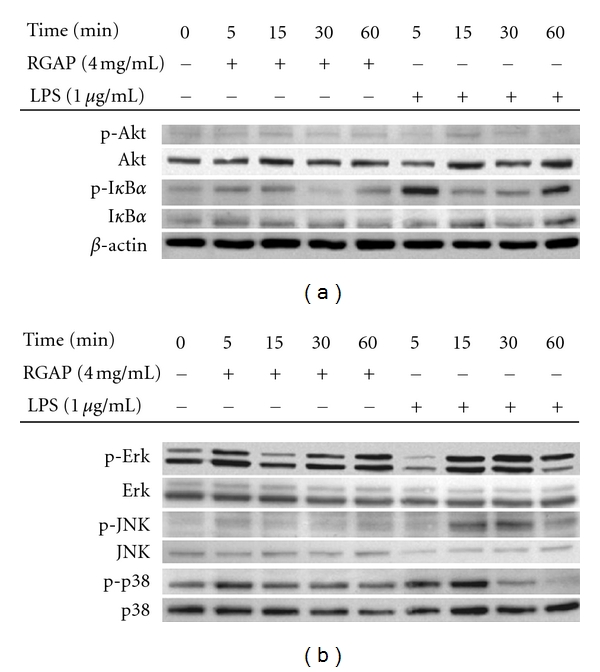
Effect of RGAP and LPS on the activation of upstream signalling enzymes for NF-*κ*B and AP-1 translocation. (a and b) Phosphorylated or total protein levels of I*κ*B*α*, Akt, ERK, JNK, p38, and *β*-actin from cell lysates prepared with RGAP- or LPS-treated RAW264.7 cells were determined by phosphospecific or total protein antibodies.

To compare the difference in transcriptional activation levels of macrophages between LPS and RGAP, intracellular signaling events were also investigated. As shown in Figure 3(a), the intracellular signaling patterns seem to be different. Namely, the inhibitory activity of tyrosine kinase inhibitors such as piceatannol, PP2, and AG490 after RGAP exposure was 2-fold less than after LPS treatment (Figure 3(a)). Interestingly, U0126, an ERK inhibitor, and SP600125, a JNK inhibitor, significantly suppressed RGAP-mediated NO production (Figure 3(a)), while these inhibitors did not block effects of LPS in RAW264.7 cells (Figure 3(b)), suggesting that JNK and ERK positively regulate RGAP-induced signaling cascades. It has been reported that MAPK plays a critical role in immunogenicity mediated by carbohydrate-containing immunogens such as *β*-glucans and lactic acid bacterium-derived peptidoglycans [34]. These data also strongly imply that intracellular signaling mechanisms are distinct between LPS and RGAP. The involvement of NF-*κ*B, AP-1, CREB, and ATF-2 in RGAP-induced macrophage activation was also demonstrated by measuring the phosphorylation levels of their upstream signaling enzymes [35]. For NF-*κ*B signaling, RGAP enhanced the phosphorylation of Akt and I*κ*B*α* within 5 to 15 min, while LPS only strongly enhanced I*κ*B*α* at 5 min (Figure 4(a)). According to our report that the phosphorylation of I*κ*B*α* at 5 min is critically regulated by Syk activity [36], Syk seems to be required for early activation of NF-*κ*B stimulated by RGAP and LPS. For MAPK activation, ERK, JNK, and p38 seemed to be activated at 5 min. In contrast, LPS-induced MAPK signaling events were distinctly seen at 15 to 30 min (Figure 4(b)). Therefore, these results strongly suggest that differentially activated molecular events in macrophage inflammatory responses by RGAP and LPS seem to differentially control the strength of immune responses triggered by RGAP or LPS. 

**Figure 5:**
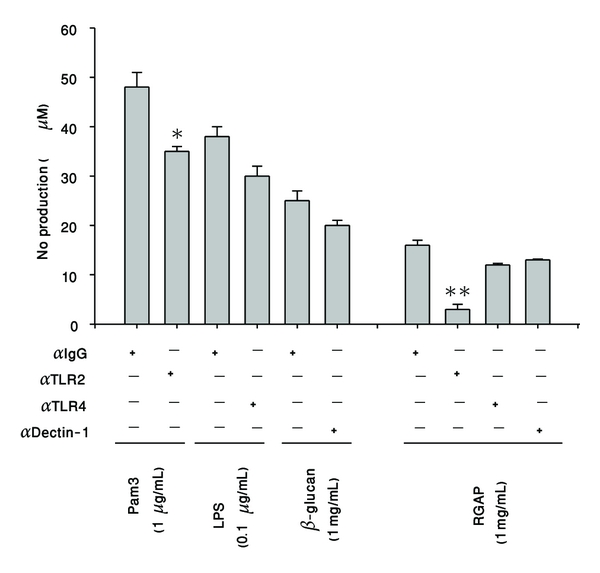
Effects of blocking antibodies on RGAP-mediated NO production in RAW264.7 cells. Levels of NO were determined by the Griess assay from culture supernatants of RAW264.7 cells pretreated with blocking antibodies to TLR2, TLR4, and dectin-1 2 h before stimulation with RGAP, pam3CSK, *β*-glucan, or LPS (1 *μ*g/mL) treatment for 24 h. *P < 0.05 and **P < 0.01 compared to control.

Finding the molecular target of RGAP is important in understanding the molecular mechanism of action for RGAP-induced immune responses. Considering that RGAP is not able to penetrate into cell membranes like other glucose moiety-containing immunogens [37], it is expected that the target of this polysaccharide is on the surface membrane. To check this, we employed several blocking antibodies to TLR2, TLR4, and dectin-1, which are known to interact with polysaccharide fractions such as *β*-glucan and Zymosan [38]. Indeed, antagonistic antibodies to TLR2, TLR4, and dectin-1 significantly suppressed NO production induced by Pam3CSK (a TLR2 ligand), LPS (a TLR4 ligand), and *β*-glucan (a dectin-1 ligand; Figure 5). Intriguingly, RGAP-induced NO production was greatly diminished by the TLR2 antibody, indicating that TLR2 could be a binding receptor for RGAP (Figure 5). Whether TLR2 antibody binds directly bind to RGAP or whether RGAP binding to TLR2 is blocked, this antibody to TLR2 requires further investigation.

**Figure 6:**
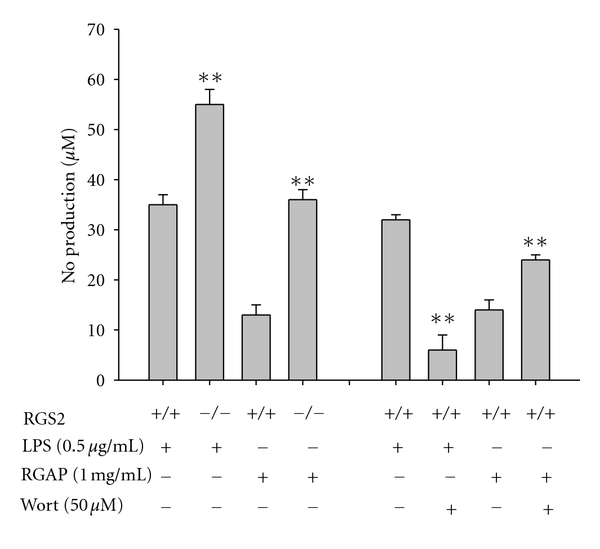
Effects of RGS2 and wortmannin on RGAP-mediated NO production in peritoneal macrophages. Levels of NO were determined by the Griess assay from culture supernatants of peritoneal macrophages prepared from wild-type or RGS2 knockout mice in the presence or absence of wortmannin, stimulated with RGAP or LPS (1 *μ*g/mL) for 24 h. *P < 0.05 and **P < 0.01 compared to control.

Finally, even though ginseng has been known to boost the body's immune responses, the stimulatory effect of ginseng polysaccharide fraction was marginal, as assessed by NO production (Figure 1). Compared to other stimulatory polysaccharides, we wish to improve the stimulatory activity of ginseng or ginseng-derived polysaccharide fractions (e.g., RGAP). In our screening experiments, we found that RGS2 and wortmannin-targeted enzyme(s) were capable of acting as negative regulators in RGAP-induced production of NO. Thus, peritoneal macrophages from RGS2 knockout mice strongly enhanced NO production up to 3-fold higher, compared with primary macrophages from wild-type mice, while LPS-induced NO production was enhanced 1.5-fold. Further, wortmannin, an inhibitor of PI3K, polo-like kinase, and myosin light chain kinase [39, 40], upregulated NO production stimulated with RGAP but not LPS (Figure 6), suggesting that a wortmannin-targeted enzyme(s) can act as a unique, negative regulator of RGAP-mediated signaling cascade linked to its immunostimulatory activity. The fact that wortmannin can act as a broad-spectrum enzyme inhibitor [39, 40] commits us to analyze which wortmannin-targeted enzyme contributes to its upregulation of RGAP-induced NO production. Knowing which enzyme involved in enhancement of this activity may enhance ginseng's immunostimulatory effects. Indeed, some components such as lucidenic acids-rich extracts have been found to improve the activity of *β*-glucans and polysaccharides from mushrooms [41]. Therefore, future work will be focused on identification of an enzyme acting as a negative regulator.

In summary, we found that RGAP treatment induced NO production in RAW264.7 cells without altering morphological changes, unlike LPS. RGAP-induced NO production was associated with enhanced levels of iNOS and nuclear transcription factors such as NF-*κ*B, AP-1, and CREB. Evaluation with specific enzyme inhibitors, phosphorylation levels of intracellular signaling proteins, and inhibitory patterns with antagonistic antibodies suggested that ERK and JNK were the most important signaling enzymes for RGAP and TLR2 may be a surface receptor for RGAP. Lastly, macrophages from RGS2 knockout mice and wortmannin exposure in RAW264.7 cells demonstrated marked upregulation of RGAP-treated NO production. Therefore, our results strongly suggest that RGAP can be used as an immunostimulatory remedy via TLR2-mediated functional activation of macrophages, which can be boosted by wortmannin-targeted enzymes. 

## Figures and Tables

**Table 1:** Sequences of primers used in real-time PCR Analysis.

Gene		Primer sequences
iNOS	F	5′-CCCTTCCGAAGTTTCTGGCAGCAGC-3′
	R	5′-GGCTGTCAGAGCCTCGTGGCTTTGG-3′
GAPDH	F	5′-CACTCACGGCAAATTCAACGGCAC-3′
	R	5′-GACTCCACGACATACTCAGCAC-3′
